# Pivotal Roles of T-Helper 17-Related Cytokines, IL-17, IL-22, and IL-23, in Inflammatory Diseases

**DOI:** 10.1155/2013/968549

**Published:** 2013-07-14

**Authors:** Ning Qu, Mingli Xu, Izuru Mizoguchi, Jun-ichi Furusawa, Kotaro Kaneko, Kazunori Watanabe, Junichiro Mizuguchi, Masahiro Itoh, Yutaka Kawakami, Takayuki Yoshimoto

**Affiliations:** ^1^Department of Anatomy, Tokyo Medical University, 6-1-1 Shinjuku, Shinjuku-ku, Tokyo 160-8402, Japan; ^2^Division of Cellular Signaling, Institute for Advanced Medical Research School of Medicine, Keio University School of Medicine, 35 Shinanomachi, Shinjuku-ku, Tokyo 160-8582, Japan; ^3^Department of Immunoregulation, Institute of Medical Science, Tokyo Medical University, 6-1-1 Shinjuku, Shinjuku-ku, Tokyo160-8402, Japan; ^4^Department of Immunology, Tokyo Medical University, 6-1-1 Shinjuku, Shinjuku-ku, Tokyo 160-8402, Japan

## Abstract

T-helper 17 (Th17) cells are characterized by producing interleukin-17 (IL-17, also called IL-17A), IL-17F, IL-21, and IL-22 and potentially TNF-**α** and IL-6 upon certain stimulation. IL-23, which promotes Th17 cell development, as well as IL-17 and IL-22 produced by the Th17 cells plays essential roles in various inflammatory diseases, such as experimental autoimmune encephalomyelitis, rheumatoid arthritis, colitis, and Concanavalin A-induced hepatitis. In this review, we summarize the characteristics of the functional role of Th17 cells, with particular focus on the Th17 cell-related cytokines such as IL-17, IL-22, and IL-23, in mouse models and human inflammatory diseases.

## 1. Introduction

**Figure 1:**
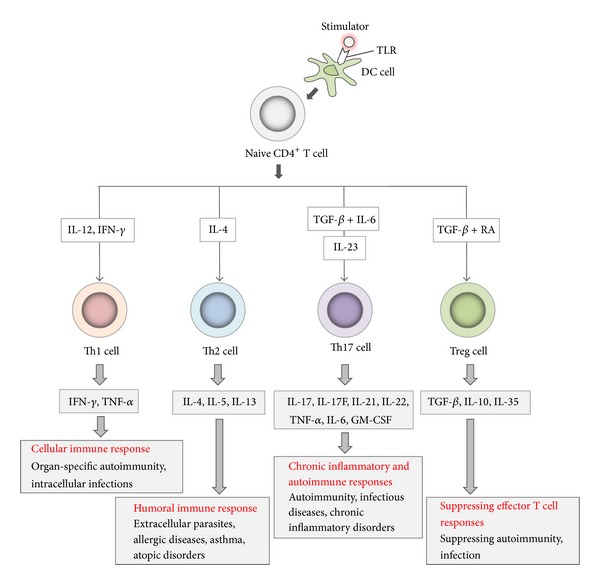
Differentiation of naïve CD4^+^ T cells. Upon certain stimulating conditions, naïve CD4^+^ T cells differentiate into different subpopulations, such as Th1, Th2, Th17, and regulatory T cells (Tregs). Th1: T-helper 1 cell; Th2: T-helper 2 cell; Th17: T-helper 17 cell; IL: interleukin; TGF-*β*: transforming growth factor-*β*; IFN-*γ*: interferon-*γ*; TNF-*α*: tumor necrosis factor-*α*; GM-CSF: granulocyte macrophage colony-stimulating factor; DC: dendritic cell; RA: retinoic acid.

CD4^+^ T-helper (Th) cells play a central role in initiating and maintaining diverse immune responses. Functionally distinct Th cells are induced when naïve T cells are stimulated via T cell receptor engagement in conjunction with costimulatory molecules and cytokines produced by innate immune cells. Classically, Th1 cells regulate cellular immunity via production of interferon (IFN)-*γ*, whereas Th2 cells regulate humoral immunity via production of interleukin (IL)-4, IL-5, and IL-13 [1, 2]. Regulatory T cells (Tregs), a third subset of CD4^+^ T cells, regulate the activation and expansion of these lineages via expression of forkhead box P3 and/or their capacity to produce cytokines such as transforming growth factor (TGF)-*β*, IL-10, and IL-35 [3, 4]. Recently, the identification of a novel lineage of helper T cells, Th17, has broken the long-held paradigm regarding the roles of the other three lineages (Th1, Th2, and Treg) (Figure 1). Distinguished by the production of IL-17 (also called IL-17A), these Th17 cells are developed from naïve CD4^+^ T cells under the influence of a network of inflammatory cytokines, including IL-1, IL-6, and TGF-*β*, which support the commitment to this lineage. Although IL-23 was previously reported to be necessary for Th17 differentiation, it is currently thought that IL-23 plays an important role in the survival and expansion of pathological Th17 cells [5–9].

 Th17 cells were first defined by their expression of IL-17A, but they have since been shown also to preferentially express IL-22, as well as IL-17F, IL-21, GM-CSF, and potentially TNF-*α* and IL-6 [10, 11]. However, it is becoming apparent that the IL-22 expression profile differs from that of IL-17A. Whereas TGF-*β* and IL-6 are both necessary for induction of IL-17A, IL-22 can be induced via IL-6 alone, and increasing amounts of TGF-*β* are actually inhibitory to the expression of IL-22 [12]. Accumulating data suggest that Th17 cells play a significant role in infectious diseases, autoimmune conditions, adoptive immune response, and mucosal immunity [13–16]. The polarization of Th17 cells relies critically upon the actions of cytokines (e.g., IL-23) secreted by antigen-presenting cells (APCs) [14, 17, 18]. In addition to the inflammatory diseases, IL-23 also plays essential roles during tumorigenesis [19].

**Figure 2:**
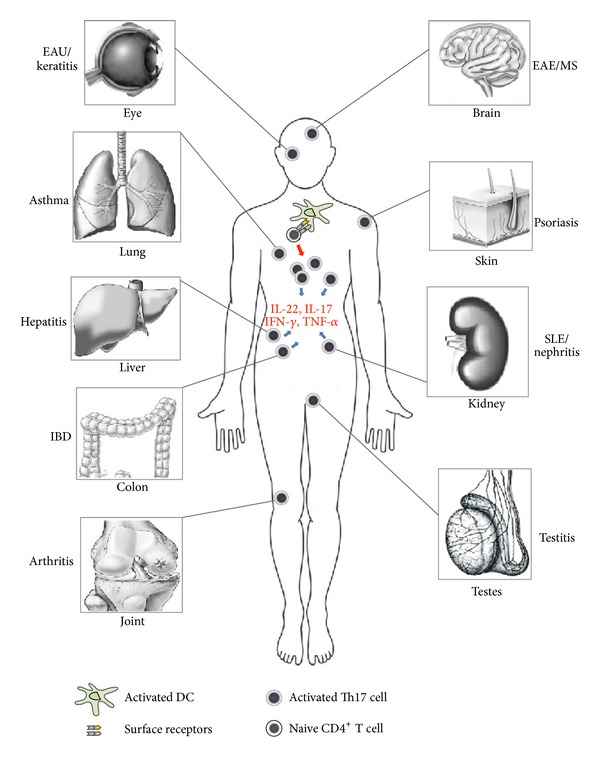
Schematic representation of Th17 cell-induced inflammatory diseases in humans. Inflammation mediated by Th17 cells has been identified in several human organs or tissues, including the eye, brain, skin, liver, colon, kidney, testes, joint, and lung. Numerous cytokines induced by activated Th17 cells, such as IL-22, IL-17, IFN-*γ*, TNF-*α*, and IL-6, play essential roles during the inflammatory diseases. These cytokines lead to the onset of the uveitis, autoimmune encephalomyelitis, psoriasis, hepatitis, inflammatory bowel disease, nephritis, testitis, rheumatic arthritis, and asthma. The counteraction between protective cytokines and pro-inflammatory cytokines decides the final outcome in the organ or tissue.

 Based on evidence that Th17 cells can mediate inflammation and tissue destruction [20, 21], there has been intense interest in defining their origins and functions and developing strategies to block their pathological effects. In this review, we highlight studies that provide significant evidence for a role of Th17 cells in human diseases and animal models, and we briefly review the role of Th17 cells by focusing on the production of cytokines in inflammatory diseases (Figure 2).

## 2. Th17 Cells in Inflammatory Skin Diseases

Inflammatory skin diseases include psoriasis, allergic contact dermatitis, and atopic dermatitis. Psoriasis is a complex autoimmune skin disease characterized by interactions between dendritic cells (DCs), T cells, and keratinocytes [22, 23]. Although mice with epidermal acanthosis and dermal inflammation induced by IL-23 injection into the ear are not an exact model for psoriasis, many of the features in this model, such as IL-22 upregulation and STAT3 activation, are similar to the features evident in psoriasis.

 In psoriasis, IL-23 is produced at high levels by DCs and keratinocytes, and this cytokine stimulates Th17 cells to produce IL-17A and IL-22. Several groups reported that psoriatic lesions showed increased mRNA levels of the IL-23/Th17 axis, including IL-23p19, IL-12/23p40, IL-22, IL-17A, and IL-17F, whereas mRNA levels of IL-12p35 and IL-4 were not elevated [24–26]. Furthermore, evidence for the role of IL-23 in the pathogenesis of psoriasis was substantiated by the initiation of the psoriasis-like disease acanthosis following repeated injections of IL-23 in mice [12]. More recent studies have also revealed that polymorphisms in the IL-12/23p40 and IL-23 receptor (IL-23R) are associated with psoriasis [27]. Ustekinumab, an anti-IL-12/23p40 antibody, has been used to treat plaque psoriasis [28]. In transgenic mice, overexpression of individual subunits of IL-23 led to inflammation [29]. In another mouse study, recombinant IL-23 injected into normal skin produced erythematous skin with histologic characteristics of psoriasis [30].

 IL-22 is a key cytokine produced by Th17 cells, and it plays an important role in maintaining homeostasis and remodeling epithelial tissues. The importance of IL-22 has been highlighted in the pathogenesis of psoriasis [12]. IL-22 mRNA expression is upregulated in psoriatic skin as compared to normal skin, whereas the levels of IL-22 mRNA in peripheral blood mononuclear cells from psoriatic patients and normal controls were similar [31]. Using IL-22-deficient mice, Zheng et al. showed that in the absence of IL-22, IL-23-mediated dermal inflammation was reduced [12]. Another study also showed that IL-22 is required for psoriasis-like lesions in the mouse Imiquimod model. Imiquimod-induced scaly skin lesions were almost totally absent in IL-22-deficient mice or in mice treated with anti-IL-22 antibody. Importantly, IL-22 mediates keratinocyte activation via phosphorylation of STAT3, leading to acanthosis that is associated with a psoriatic phenotype [12, 32].

 In addition, injection of IL-23 enhances IL-17A expression in mouse skin, but pretreatment of anti-IL-17A antibody does not ameliorate the formation of psoriatic lesions [30]. This observation suggests that IL-17A is dispensable during IL-23-dependent psoriasis. Skin biopsy samples from patients with psoriasis showed elevated expression levels of IL-17 together with high expression of IL-23 and IL-22 [12, 33]. Although there was no difference between the levels of IL-17 in the sera of patients with psoriasis and in controls, there was a correlation between serum levels of IL-17 and the severity of psoriasis [34].

## 3. Th17 Cells in Inflammatory Bowel Diseases

Inflammatory bowel disease (IBD), including Crohn's disease and ulcerative colitis, is a chronic inflammatory disease of the gastrointestinal tract. IBD is caused by aberrant innate and/or adaptive immune responses [35]. IBD had long been described as a Th1-mediated disease because IFN-*γ* is essential for disease progression [36]. However, the recent discovery of Th17 cells has revealed a key role of this subset of T cells in IBD.

 IL-23 is essential for the development of IBD in mouse models [37, 38], and protective IL-23R polymorphisms in the human population were identified through a genome association study [39]. However, IL-22 stimulates epithelial cell growth, goblet cell hyperplasia, and antimicrobial production. IL-22-mediated protective effects were seen in the T cell transfer colitis model [40]. IL-22 is highly upregulated in the sera and lesions of patients with either Crohn's disease or ulcerative colitis [41]. Moreover, activation of aryl hydrocarbon receptor (AHR) results in the elevated production of IL-22 in particular and reduction of Th1 and Th2 cytokines [42, 43]. Blockade of IL-22 by using its neutralizing antibody reversed the therapeutic effect of 6-formylindolo (3, 2-b) carbazole on the trinitrobenzenesulfonic acid-induced colitis in mice. Thus, induction of IL-22 is one of the major mechanisms controlling pathogenesis in the gut through the AHR signaling pathway [44].

 On the other hand, IL-17 is produced in healthy gut. A recent study suggested that IL-17F, but not IL-17A, was required to induce severe immunopathology in the dextran sulfate sodium-induced colitis model [45]. In contrast, anti-IL-17A monoclonal antibody treatment was demonstrated to aggravate dextran sulfate sodium-induced colitis, and blockade of IL-17A in colitis of IL-10 knockout mice was inefficient in reducing disease unless IL-6 was also neutralized [46, 47]. Another study demonstrated that adoptive transfer of IL-17A-deficient naïve CD4^+^ T cells or transfer of IL-17 receptor-deficient T cells to recipient immunedeficient mice induces severe colitis [48], suggesting that IL-17 exerts a protective effect on T cells. Collectively, these results indicate that Th17 cytokines have both anti- and pro-inflammatory effects in the gut, depending on the microenvironments.

## 4. Th17 Cells in Experimental Autoimmune Encephalomyelitis/Multiple Sclerosis

Experimental autoimmune encephalomyelitis (EAE), which resembles an autoimmune inflammatory disease of human multiple sclerosis (MS), was classically believed to be mediated by Th1 cells and inflammatory macrophages. However, the concept that Th1 response is centrally important for autoimmunity was challenged by evidence that animals lacking a functional Th1 response still develop aggravated autoimmune encephalomyelitis. Recent studies demonstrated an association between the development of demyelinating plaques and the accumulation of Th17 cells in EAE and MS.

 IL-23 plays a pivotal role in the development of EAE. Mice deficient in IL-23p19 or IL-23R knockout mice were resistant to EAE [5, 49, 50]. Moreover, IL-23R is expressed in macrophages infiltrating the central nervous system, and macrophages expressing IL-23R in response to IL-23 produce IL-22 and IL-17 [5, 50].

 In addition, IL-17 (IL-17A) also plays a pro-inflammatory role during the development of EAE, as shown by several lines of evidence. First, IL-17F knockout mice with normal levels of IL-17A showed only marginally reduced EAE [45]. Second, IL-17A knockout mice with normal levels of IL-17F showed milder disease [51]. Finally, IL-17A knockout mice with reduced levels of IL-17F exhibited clearly reduced EAE [15]. Moreover, administration of anti-IL-17A antibody could attenuate EAE but not completely prevent this disease [52].

 Although IL-22 can be induced from Th17 cells by IL-23 during inflammation, IL-22 seems to have no effect on the development of EAE. Kreymborg et al. showed that IL-22 knockout mice are not protected from EAE [53].

 In MS patients, IL-17 mRNA and protein levels were increased in both brain lesions and mononuclear cells isolated from blood and cerebrospinal fluids [54, 55]. Although these observations suggest that IL-17 may contribute to the development of MS in humans, further research is needed to elucidate the precise role of this cytokine in the pathogenesis of MS. In addition, because IL-23 plays a pivotal role in EAE, administration of monoclonal antibody specific for IL-23p19 instead of IL-17A or IL-17F might prevent this disease [56]. Based on these results, neutralization of IL-23 may be an effective therapeutic approach to treat EAE/MS.

## 5. Th17 Cells in Rheumatoid Arthritis

Rheumatoid arthritis (RA) is a chronic inflammatory disease associated with the destruction of affected joints, and it represents one of the most common autoimmune-related diseases. Although RA had long been classified as a Th1-mediated disease, it is now thought to be a primarily Th17-driven disease [57].

 Initial evidence for a pathogenic role of IL-17 in RA came from reports that IL-17 was increased in the sera and synovial fluids of RA patients [58–60]. Long-term intra-articular administration of IL-17 via gene transfer reproduced the key features of RA, including massive inflammation, bone erosions, and cartilage damage [61]. As with psoriasis, there is also increased IL-22 and IL-23 in the synovium of RA patients [62, 63]. Notably, the increase in IL-17 and IL-23 appears to be specific for RA, but not for osteoarthritis [64, 65]. Conversely, inhibition of IL17 by antibodies against IL-17A or its receptor IL17RA protected against the development of arthritis [66]. Because cyclosporine A can inhibit the production of IL-17 by memory Th17 cells in healthy donors and RA patients [67], this could be an effective strategy to limit the disease. Furthermore, mice lacking IL-17RA develop a very mild form of experimental arthritis [68]. TNF has been shown to be a key cytokine in the collagen-induced arthritis model. Although TNF contributes to the pathogenesis of the early stages of the disease, it is not involved in the later stages. In contrast, IL-17 has a role throughout all stages of chronic disease [69]. This finding is another indication that IL-17 contributes to the chronicity of RA. Therapeutic strategies that specifically block Th17 cell development are expected to be highly effective in treating RA patients.

## 6. Th17 Cells in Renal Inflammation

Several recent studies have emphasized the functional importance of Th17-induced immune response in renal inflammatory diseases. We discuss the potential roles of the Th17 immune response in experimental murine models and humans.

### 6.1. Th17 Cells in Experimental Animal Models with Nephritis

The first evidence for the importance of TH17 cells in renal inflammation was provided by a murine model of crescentic glomerulonephritis [70, 71]. Recently, Th17 cells were identified in murine kidneys after ureteral obstruction [72]. In addition, the IL-23/IL-17 pathway was demonstrated to contribute significantly to renal tissue injury in experimental glomerulonephritis by analysis of nephrotoxic nephritis in both IL-23p19 and IL-17 knockout mice [70]. Moreover, IFN-*γ* plays a protective role in experimental autoimmune anti-glomerular basement membrane (anti-GBM) glomerulonephritis, as revealed by the fact that IFN-*γ*-deficient mice develop more severe anti-GBM disease [73]. In contrast, IL-23p19 and IL-17A knockout mice are protected from anti-GBM disease after treatment with anti-mouse GBM antibodies [70]. In addition, by using IL-12p35, IL-12p40, and IL-23p19 knockout mice, Ooi et al. demonstrated that mice deficient in IL-23, but not IL-12, were protected from glomerulonephritis [74]. Neutrophils were recently identified to be an early source of IL-17 in renal inflammation in a mouse kidney ischemia reperfusion injury model [75].

### 6.2. Th17 Cells in Human Renal Inflammation

There is only limited evidence of the involvement of Th17 cells/IL-17 in the pathogenesis of renal autoimmunity in humans. The contribution of IL-17 to inflammatory reactions in the kidney was initially reported in an *in vitro* study of patients suffering from renal transplantation graft rejection [76]. Recently, upregulation of IL-17 mRNA expression in the urinary sediment of patients with systemic lupus erythematosus (SLE) and increased percentage of Th17 cells in patients with active SLE were reported [77, 78]. Although serum IL-17 levels were significantly increased in SLE patients compared with normal controls, associations between serum IL-17 levels and clinical parameters were demonstrated [79]. Another study reported that a lower percentage of Th22 cells and higher percentage of Th17 cells are present in patients with lupus nephritis compared with healthy controls [80]. Th22 cells are a new subset of CD4^+^ T helper differentiated from naïve T cells and characterized by secretion of IL-22 but not IL-17 or IFN-*γ* [43, 81]. IL-22 may play a protective role in preventing the development of lupus nephritis, although future research is necessary to identify the real role of IL-22 in SLE.

## 7. Th17 Cells in Hepatitis

### 7.1. Th17 Cells in Experimental Hepatitis Models

Intravenous administration of Concanavalin A (Con A) results in rapid liver inflammation and necrosis [82]. Many features of Con A-induced liver injury are believed to mimic human autoimmune and viral liver disorders. Numerous experiments have also shown that IL-22 plays a protective role in mice with hepatitis [83, 84]. However, there are conflicting reports regarding the susceptibility of *IL-17*-deficient mice to Con A-induced acute hepatitis [84, 85]. IL-17 is critical in the induction of liver injury and is induced during Con A hepatitis [86, 87]. Moreover, both IL-17A and IL-17F function via the IL-17 receptor (IL-17R), and both *IL-17A* and *IL-17F* are overexpressed in IL-17R-deficient mice, suggesting that a feedback loop acts on Th17 cells [88, 89]. In addition, IL-17 activates other cell types in the liver to produce pro-inflammatory cytokines beneficial to hepatocyte apoptosis [90].

**Figure 3:**
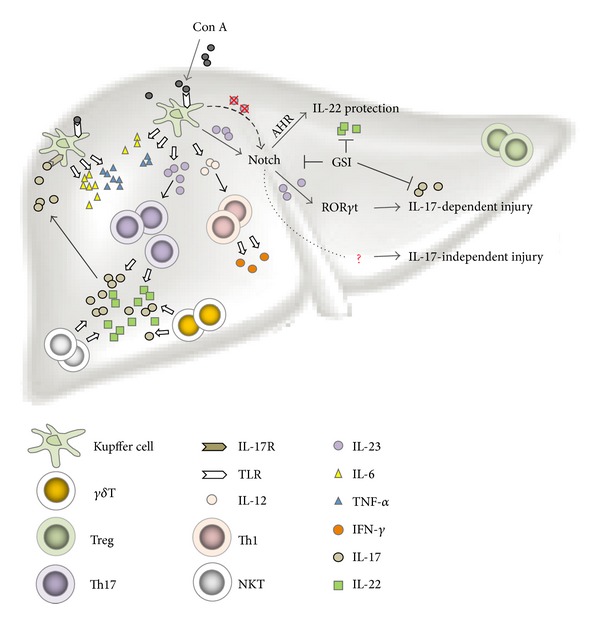
Schematic diagram of the role of activated Th17 cells during Con A-induced hepatitis. Con A injection induces IL-23 expression from Kupffer cells (also inducing IL-12, IL-6, TNF-*α*, and other cytokines) in the liver, then activates Notch signaling in activated Th17 cells (and other types of T cells). AHR-dependent production of IL-22 is pivotal for protection, and ROR*γ*t-dependent production of IL-17 is critical for pathogenesis. The IL-17/IL-17R signaling pathway also exacerbates hepatitis by inducing TNF-*α* and IL-6. Con A: Concanavalin A; AHR: aryl hydrocarbon receptor; ROR*γ*t: retinoic acid-related orphan receptor *γ*t; TLR: toll-like receptor; GSI: *γ*-secretase inhibitor; IL-17R: interleukin-17 receptor.

 Notch is an evolutionarily conserved molecule that controls the cell fate decision in a variety of cells [91, 92]. We previously demonstrated that Notch signaling drives IL-22 secretion by stimulating the AHR [93]. Mice that are deficient in RBP-J, a key mediator of Notch signaling, are highly susceptible to the detrimental immunopathology associated with Con A-induced hepatitis with little IL-22 production [93] (Figure 3). Although IL-6 has the ability to induce IL-22 production [12], and IL-6-deficient mice were shown to be highly susceptible to liver damage [94], these mice were reported to have no impairment in IL-22 expression during Con A-induced hepatitis [84]. IL-23 also has the ability to induce the production of IL-22 [12] and IL-17 [95]. However, there are conflicting reports regarding the role of IL-17 in Con A-induced hepatitis and the susceptibility of IL-17-deficient mice to hepatitis [84, 85]. Therefore, the role of IL-23 in the induction of IL-22 and IL-17 production and liver damage during Con A-induced hepatitis using IL-23p19- and IL-17-deficient mice was investigated [86]. These results revealed that the endogenous IL-23 plays a protective role in hepatitis in an IL-22-dependent manner, whereas exogenous IL-23 plays a pathological role in IL-17-dependent and -independent manners. Further studies are necessary to elucidate the precise role of exogenous IL-23 in Con A-induced hepatitis.

### 7.2. Th17 Cells in Human Liver Diseases

Chronic hepatitis B virus (HBV) or hepatitis C virus infection leads to liver disease. Such infective disease is associated with T cell activation and the secretion of numerous pro-inflammatory cytokines, such as IFN-*γ*. Nonetheless, although IL-22 shows a marked protective role in Con A-induced hepatitis, IL-22 also enhances the pro-inflammatory activity of TNF-*α* expressed in the liver after transfer of HBV-specific T cells [96]. Another study reported that IL-22 neutralization ameliorates liver damage after transferring HBV-specific T cells by using a transgenic mouse model of HBV replication [97]. During acute liver inflammation, IL-22 protects hepatocytes from injury, possibly through STAT3-mediated upregulation of prosurvival and proliferative responses. During chronic inflammation, IL-22 may also help to limit damage and allow survival of damaged hepatocytes that are precursors for hepatocellular carcinomas [98]. Future research is necessary to examine the role of IL-22 in chronic inflammation and the development of liver cancer. In patients with chronic HBV infection, Th17 cells are highly increased in both peripheral blood and liver, and they exhibit a potential to aggravate liver damage during chronic HBV infection [99]. Thus, Th17 cells may be involved in both the pathogenesis and anti-inflammatory responses in human liver diseases.

## 8. Th17 Cells in Ophthalmic Inflammation

### 8.1. Th17 Cells in Experimental Autoimmune Uveitis

The eye is an immune-privileged organ, and immune privilege is a complex phenomenon that involves multiple components. Uveitis is a sight-threatening intra-ocular inflammatory disease that is predominantly mediated by Th1 and Th17 [100]. Experimental autoimmune uveitis (EAU) is an animal model of human autoimmune uveitis, and activated Th1 and Th17 cells are considered to play a major role in initiating the intra-ocular inflammation [101].

 The initial evidence indicated that Th1 cells predominantly produce IFN-*γ* in experimental and clinical uveitis [102–104]. However, it is now clear that IL-17-producing Th17 cells, but not IFN-*γ*-producing Th1 cells, are the true mediators of tissue-specific ocular pathogenesis [81, 105]. Neutralization of IL-17, but not IFN-*γ*, in mice prevents and ameliorates EAU [104, 106]. Several recent studies suggested that IL-17 has both pro- and anti-inflammatory effects on the development of EAU [107, 108]. Furthermore, a protective role of IL-22 by inducing regulatory CD11b^+^ APCs has been described in EAU [109]. In addition, CD4^+^ T cells are necessary for initiating EAU, and depletion of CD4^+^ T cells prevents EAU development. Furthermore, antigen-specific CD8^+^ T cells also act as regulatory cells to suppress EAU [110, 111]. Similar to other autoimmune animal models, costimulatory signals such as CD40, CD80, and CD86 are also involved in the course of EAU, and blockade of these signals ameliorates intra-ocular inflammation [112–116].

### 8.2. Th17 Cells in Keratitis

IL-17A-producing cells are present in the midperipheral cornea in a mouse model of dry eye disease, as well as in corneas from patients with herpetic stromal keratitis (SK) [117–119]. The cornea infection with herpes simplex virus (HSV) 1 leads to SK, a blinding immune-inflammatory lesion of the eye. IL-17 is upregulated after HSV infection of the cornea [120]. HSV infection of IL-17R knockout mouse as well as IL-17 neutralization in wild-type mouse showed reduced SK damage [120]. In addition, administration of 2,3,7,8-tetrachlorodibenzo-p-dioxin, which is a ligand for AHR, caused a significant induction of Tregs and inhibited the differentiation of Th1 and Th17 cells, resulting in suppression of the severity of SK damage [121].

*Staphylococcus aureus* and *Pseudomonas aeruginosa* often cause bacterial keratitis, and these bacteria predominantly invade corneal epithelial cells [122]. IL-6, one of the major cytokines responsible for differentiating into Th17 cells, is expressed in the corneal epithelial and conjunctival cell lines [123]. Desiccating stress in the murine dry eye model, similar to human dry eye, also causes ocular surface inflammation characterized by increasing IL-6 and IL-17A expression [118]. In general, IL-17RA is constitutively expressed in cornea and conjunctiva. When infected with *S. aureus*, human corneal epithelial cells were demonstrated to increase the production of IL-6 but show no change of IL-17A and IL-17RA *in vitro* [124].

## 9. Th17 Cells in Testes

The testis is an immunologically privileged site where germ cell antigens are protected from autoimmune attack [125–128]. Multiple mechanisms prevent autoimmune disease in the testes, including the structure of the blood-testis barrier and secretion of immunosuppressive factors mainly by macrophages, Sertoli, peritubular, and Leydig cells. Studies established the presence of several T cell subsets (CD4^+^ and CD8^+^ *αβ* T cells, *γδ* T cells, and NK cells) and Tregs, as modulators of immune response acting through local and systemic mechanisms, in normal testicular interstitium of human and rodents [129, 130]. However, the testicular environment does not preclude inflammatory reactions and recruitment of tissue-specific T lymphocytes, which appear to be crucial components of the inflammation cascade [131, 132]. In fact, testicular inflammatory disorders leading to impairment of spermatogenesis are thought to be a primary reason for male infertility [133–135]. The recruitment of immune cells in testicular interstitium (mainly DCs, macrophages, and T cells) and secretion of pro-inflammatory cytokines (IL-6, IFN-*γ*, TNF-*α*, IL-12, IL-17, and IL-23), which disrupt the normal testicular immunosuppressive microenvironment, occur during inflammation induced by infectious agents or develop in different pathologies, such as experimental autoimmunity orchitis (EAO) [136]. In the rat testis of EAO, it was demonstrated that not only CD4^+^ cells (Th17) but also CD8^+^ T cells produce IL-17 (Tc17). Both CD4^+^ and CD8^+^ T cells are the major contributors during the onset and chronic phases of EAO [136]. In human azoospermic testis with chronic inflammation, Th17 cells, which are orchestrated by IL-23 produced from APCs, are critically involved in chronic inflammation [137]. Such patients have increased levels of Th17 cells, their cytokines such as IL-17A, IL-21, and IL-22, and IL-23-producing CD11c^+^ DCs and CD68^+^ macrophages [137]. Moreover, because IL-17 was expressed not only in normal testis but also in higher levels in azoospermic testis, IL-17 might be involved in the maintenance of testicular immune privilege and spermatogenesis [137, 138]. In addition, pro-inflammatory cytokines including IL-1 and IL-6 have direct effects on spermatogenic cell differentiation and testicular steroidogenensis within the normal testis [139]. However, increased numbers and expression level of IL-17A-immunoreactive cells in azoospermic testis with chronic inflammation indicate that overexpression of IL-17A can substantially damage the blood-testis barrier and probably destroy normal spermatogenesis and germ cells, which in turn could ultimately lead to azoospermia.

 IL-17-deficient mice showed decreased antigen-specific T cell activation and antibody production in models of autoimmune and allergic diseases [140]. In addition to the signature cytokine IL-17A (IL-17), Th17 cells also produce IL-17F, IL-21, and IL-22, which would also allow Th17 cells to communicate with a wide variety of immune and non-immune cells [14]. A recent study demonstrated that the small molecule halofuginone can selectively inhibit mouse and human Th17 cell differentiation and autoimmune inflammation *in vivo* through a cytoprotective signaling pathway [141]. An understanding of the development, function, and regulation of Th17 cells in testicular immunopathology is critical for designing better strategies for the treatment of immunological male infertility.

## 10. Th17 Cells in Allergic Airway Disease

Asthma is characterized by an inflammatory reaction associated with increased production of Th2 type cytokines, such as IL-4 and IL-13.

### 10.1. Th17 Cells in Mouse Models

Numerous studies have shown that Th17 cytokines play an essential role in allergic airway disease, and the role of Th17 cells was investigated in several mouse models. A study of IL-17RA knockout mice demonstrated decreased ovalbumin-induced airway eosinophilia and Th2-related cytokines [142]. IL-17A knockout mice showed attenuated airway eosinophilia and neutrophilia, whereas IL-17F knockout mice demonstrated elevated eosinophil recruitment. These findings suggest that IL-17 drives the allergic Th2 response. Other studies also confirmed that IL-17 promotes ovalbumin-induced Th2 responses by synergizing with IL-4 and IL-13 [143]. Unlike IL-17, which has a pro-inflammatory role during allergic airway disease, IL-22 seems to suppress Th2-mediated inflammation. Treatment with anti-IL-22 antibody exacerbated airway eosinophilia, suggesting that IL-22 may have anti-inflammatory properties in airway disease [144]. In contrast, IL-23 knockout mice showed ameliorated eosinophilia compared to IL-23 overexpression mice [145, 146].

### 10.2. Th17 Cells in Human Asthma

The role of Th17 cells in human asthma remains largely elusive. In humans, increased expression of IL-17A and IL-17F was detected in bronchial submucosa, and examination of sputum in patients with asthma demonstrated that neutrophils were present, particularly in severe forms of this disease [147]. Furthermore, Lajoie et al. demonstrated a direct link among C5aR signaling, IL-17A production, and severe airway hyperresponsiveness; the sensitivity of airway hyperresponsiveness noted in mice after C5aR blockade is completely reversed by concurrent IL-17A blockade [148]. In addition, polymorphisms in the *IL-17A* gene related to asthma risk have been reported [149]. Further studies are necessary to clarify whether IL-17 is a safe therapeutic target for asthma therapy.

## 11. Conclusion

Th17 cells, which are directly involved in and mediate chronic inflammation, are characterized by the production of cytokines such as IL-17 and IL-22 as well as the recruitment of neutrophils and other inflammatory cells. Under certain circumstances, the same cytokine plays opposite roles in different tissues. For instance, IL-22 plays a protective role in Con A-induced acute hepatitis but a pro-inflammatory role in psoriasis. In different tissues, the counteraction between protective cytokines and pro-inflammatory cytokines should determine the final outcome of the immune responses. Although some conflicting findings still need to be resolved, targeting Th17 cells and their related cytokines such as IL-17, IL-22, and IL-23 may be an effective therapeutic approach for chronic inflammation in the future.
